# Immersive Surgical Anatomy of the Frontotemporal-Orbitozygomatic Approach

**DOI:** 10.7759/cureus.6053

**Published:** 2019-11-02

**Authors:** Roberto Rodriguez Rubio, Ricky Chae, Ioannis Kournoutas, Adib Abla, Michael McDermott

**Affiliations:** 1 Neurological Surgery, University of California, San Francisco, USA

**Keywords:** surgical techniques, volumetric models, extended reality, immersive, surgical neuroanatomy, subfascial, intrafascial, orbitozygomatic approach, orbito-pterional approach, mini-orbitozygomatic

## Abstract

The frontotemporal-orbitozygomatic (FTOZ) approach is widely used for accessing anterolateral lesions in skull base surgery. Many studies have described the technique and quantified the surgical exposure and freedom provided by the FTOZ approach. However, few studies have provided a detailed analysis of the technique and surgical landmarks using three-dimensional (3D) models. In this study, we aimed to create a collection of volumetric models (VMs) and stereoscopic media on the step-by-step surgical technique of the FTOZ approach using cadaveric dissections. The FTOZ approach was divided into eight major steps: positioning, incision of the skin, dissection of scalp flap, mobilization of the temporalis muscle, dissection of periorbita, craniotomy, drilling of basal structures, and dural opening. The MacCarty keyhole and inferior orbital fissure are major surgical landmarks that were referenced for the six bony cuts. Photogrammetry and structured light scanning were used to construct high-resolution VMs. We illustrated the two-piece FTOZ craniotomy, followed by the one-piece and three-piece FTOZ craniotomies. Stereoscopic images, videos, and VMs were produced for each step of the surgical procedure. In addition, the mini-orbitozygomatic (MOz) and orbitopterional (OPt) approaches were considered and described as possible alternatives to the FTOZ approach. Recent advances in 3D technology can be implemented in neurosurgical practice to further enhance our spatial understanding of neurovascular structures. Surgical approaches should be carefully selected and tailored according to the patient’s unique pathology and needs.

## Introduction

As a neurosurgical technique, the frontotemporal-orbitozygomatic (FTOZ) approach is an extension of the standard pterional approach (PA), and it provides wide exposure of the orbit and anterior, middle, and posterior cranial fossae. The FTOZ approach was popularized by Pellerin et al. and Hakuba et al. in the 1980s to access parasellar and interpeduncular lesions with minimal brain retraction in skull base surgery [1,2]. In the FTOZ approach, similar to the pterional approach, the superficial layers of the frontotemporal region are carefully dissected to preserve the temporal branches of the facial nerve (TBFN) and mobilize the temporalis muscle. However, the FTOZ approach involves dissection of the periorbita to elevate the zygoma, orbital roof, and superior and lateral walls of the orbit as a second bone flap. The removal of this orbitozygomatic bone piece ensures maximum exposure of intradural structures while improving cosmetic outcomes and eliminating the need for additional bone reconstruction around the orbit. In particular, the FTOZ approach has allowed for a greater working area and angle of attack compared to the pterional approach [3]. Through the use of volumetric models (VMs) and stereoscopic media, this article aims to demonstrate a three-dimensional (3D) orientation of surgical landmarks and techniques of the FTOZ approach.

## Technical report

Materials and methods

Five embalmed and latex-injected cadaveric heads were prepared for surgical simulation along with two dry-skulls, which were used to identify and document the relevant osseous structures of the FTOZ. Dissections were performed under a surgical simulation setting using a surgical microscope (OPMI Pentero 900, Carl Zeiss AG, Oberkochen, Germany), and stop-motion frames were recorded with a high-definition stereoscopic video device (Truevision 3D, Truevision Systems, Goleta, CA). Stereoscopic (side-by-side) pictures were taken using a professional camera (D810, Nikon, Tokyo, Japan), and selected specimens were prepared for two 3D scanning techniques (i.e., photogrammetry and structured light scanning). Our laboratory had previously documented the comprehensive workflow for both of these methods [4]. No IRB/ethics committee approval was required for this study.

Virtual Platform

The models were uploaded to a web-based 3D model viewer app (Sketchfab, Sketchfab Inc, New York, NY), a platform that belongs to a series of new modalities meant to enhance the immersive and functional capacities of VMs. Once the VMs were uploaded, the virtual scenario was prepared for its real-time rendering. Position, lighting, materials, and filters were set to highlight regions of anatomical interest. Strategic points were labeled and annotated for an interactive experience. Views of the models were set for both 2D and 3D experiences.

Indications

The FTOZ approach exposes structures within and in the vicinity of the cranial fossae (the basilar artery apex, orbital apex, petrous apex, upper clivus, infratemporal fossa, interpeduncular fossa, pterygopalatine fossa, and sellar/parasellar regions) and allows higher maneuverability than the PA [5,6]. Therefore, this approach may be used for several pathologies, such as pituitary macroadenomas, chordomas, sphenoid wing meningiomas, orbital meningiomas, petroclival meningiomas, trigeminal neurinomas, basilar tip aneurysms, anterior communicating artery aneurysms, and lesions of the cavernous sinus [7,8]. Preoperative assessment of the patient’s pathology and medical history are essential for understanding the anatomy of the lesion and tailoring the surgical approach to fit the needs of each case.

Anatomical Considerations

A precise understanding of the bone, cranial sutures, and soft tissue layers is necessary to identify important landmarks and carefully navigate through the areas of exposure provided by the FTOZ approach (Interactive Model 1-3). This will enable the surgeon to increase the efficiency of the procedure and minimize the risk of cosmetic and functional complications for the patient. The areas of exposure can be divided into four parts: anterolateral, intraorbital, extracranial, and intracranial. 

**Video 1:** Volumetric model of the skull, with annotations of major ectocranial landmarks The following instructions can be used to manipulate all models: to move, left click and drag; to zoom in and out, use the mouse scroll. For smartphones and virtual reality (VR)-ready computers, click “view in VR” (glasses icon); to view annotations, click on the numbers; to move around the object, tap or press trigger on the floor using the blinking yellow circle as a pointer. For mobile augmented reality (AR), click on the AR icon (cube) in the top right corner and aim at a horizontal flat surface; once the surface is detected, tap on it to place the model

**Video 2:** Volumetric model of surgical anatomy, including soft tissue layers, involved in the frontotemporal-orbitozygomatic (FTOZ) approach The left side shows a multi-layer dissection of the galea, temporal fascia, and pericranium. The right side shows vascular structures below the zygoma and within the orbit. The course of the superficial temporal artery and temporal branches of the facial nerve can be observed bilaterally

**Video 3:** Volumetric model of the frontotemporal-orbitozygomatic (FTOZ) window The FTOZ approach provides access to basilar artery apex, orbital apex, petrous apex, upper clivus, infratemporal fossa, interpeduncular fossa, pterygopalatine fossa, and sellar/parasellar regions

**Figure 1:**
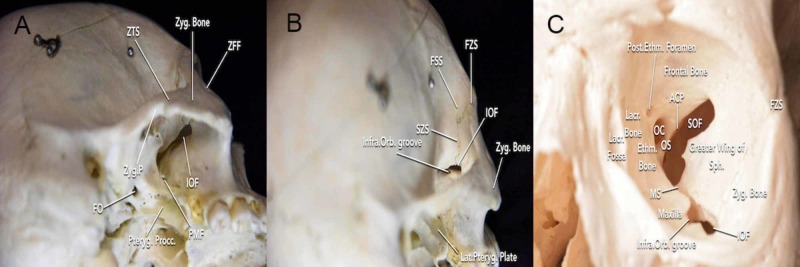
Visualization of the bone anatomy surrounding the inferior orbital fissure (A) inferolateral perspective of the skull; (B) posterolateral perspective of the skull; (C) external view of orbit ACP = anterior clinoid process; Ethm = ethmoidal; FO = foramen ovale; FSS = frontosphenoidal suture; FZS = frontozygomatic suture; SON = supraorbital notch; OC = optic canal; OF = optic foramen; OS = optic strut; SOF = superior orbital fissure; Infra.Orb.groove = infraorbital groove; IOF = inferior orbital fissure; Lacr = lacrimal; Lat.Pteryg.plate = lateral pterygoid plate; MS = maxillary strut; PMF = pterygomaxillary fissure; Pteryg.Procc = pterygoid process; Sph = sphenoid; SZS = sphenozygomatic suture; ZFF = zygomaticofacial foramen; ZTS = zygomaticotemporal suture; Zyg = zygomatic; Zyg.P = zygomatic process

In the anterolateral region, one of the first bony landmarks encountered is the frontozygomatic suture. The frontozygomatic suture can be followed medially to identify the junction of the frontozygomatic, sphenofrontal, and sphenozygomatic sutures. This three-suture junction will be important for accurately placing the MacCarty keyhole. In addition, the sutures can be used to locate intraorbital landmarks that are referenced during the osteotomy, including the inferior orbital fissure (IOF) and superior orbital fissure (SOF). For instance, the sphenozygomatic suture can be followed inferiorly from the three-suture junction to reach the IOF, and the frontozygomatic suture can be followed medially from an intraorbital perspective to reach the superolateral portion of the SOF. The IOF can also be visualized extracranially through the superior and medial portions of the infratemporal fossa (Figure 1). 

**Figure 2:**
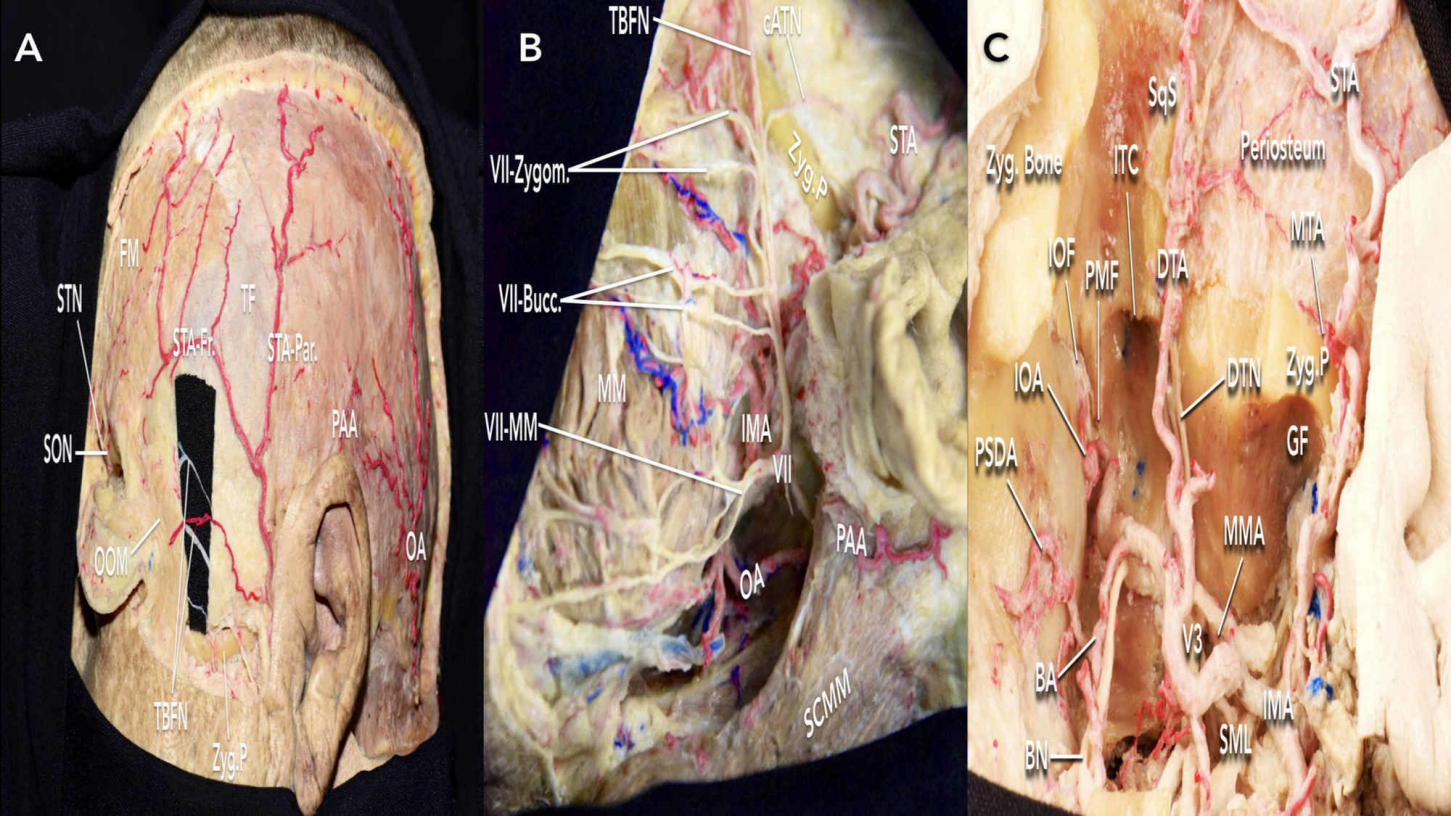
Arteries and nerves relevant to the frontotemporal-zygomatic (FTOZ) approach (A) arteries of the supra and infra zygomatic region; (B) facial nerve anatomy near incision site to be considered; (C) facial nerve anatomy superior to the zygomatic process BA = buccal artery; BN = buccal nerve; cATN = communicating auriculotemporal nerve; DTA = deep temporal artery; DTN = deep temporal nerve; FM = frontalis muscle; GF = glenoid fossa; IMA = internal maxillary artery; IOA = infraorbital artery; ITC = infratemporal crest; MM = masseter muscle; MTA = middle temporal artery; PMF = pterygomaxillary fissure; PSDA = posterior superior dental artery; SML = sphenomandibular ligament; SON = supraorbital nerve; STN = supratrochlear nerve; SqS = squamous suture; V3 = mandibular nerve; VII Zygom. = zygomatic branches of the facial nerve; VII-Bucc. = buccal branches of the facial nerve; VII-MM = marginal mandibular branch of the facial nerve; VII-Cerv. = cervical branch of the facial nerve; OA = occipital artery; OOM = orbicularis oculi muscle; PAA = posterior auricular artery; SCMM = sternocleidomastoid muscle; STA-Fr = frontal branch of the superficial temporal artery; STA-Par = parietal branch of the superficial temporal artery; TBFN = temporal branches of the facial nerve; TF = temporal fascia; Zyg = zygomatic; Zyg.P = zygomatic process

The zygomaticofacial foramen is the anteroinferior limit of the FTOZ osteotomy that serves as a landmark for the cut across the zygoma, above the malar eminence, and into the lateral edge of the IOF [3]. It is located in the lateral surface of the zygomatic bone close to the orbital rim and transmits the zygomaticofacial nerve, a branch of the maxillary nerve (V2). However, the zygomaticofacial foramen may be entirely absent or present as multiple foramina, which reduces its usefulness as a surgical landmark. Furthermore, the transverse facial artery is a branch of superficial temporal artery (STA) that courses laterally to the zygomatic arch and then anteriorly toward the lateral orbital rim. When dissecting the zygoma, utmost care should be taken to avoid injuring this artery (Figure 2A).

Intracranially, special attention should be given to the meningo-orbital band (MOB) that lies in the superolateral aspect of the optic canal. The MOB is a dural fold that binds the frontotemporal basal dura to the periorbita via the SOF. For most parasellar lesions, the MOB is the medial limit of drilling. However, if an extradural anterior clinoidectomy is necessary, the MOB can be safely detached by peeling the dura propria from the temporal lobe [9].

Surgical technique

Positioning

The patient is positioned supine, with the head rotated about 30 degrees to the side opposite to the lesion and secured using a three-pin skull fixation device (e.g., Mayfield, Mizuho OSI, Union City, CA). The two contralateral pins should be set on the superior temporal line (STL) and the ipsilateral pin on the mastoid process. The head may be rotated further, depending on the location of the lesion. In addition, the neck is slightly extended to make the malar eminence the highest point in the surgical field. This allows for a natural separation of the frontal lobe from the orbital roof and improves the angle of the microsurgical view from inferior to superior.

Incision of the Skin

A curvilinear skin incision begins at 1 cm anterior to the tragus and extends to the contralateral midpupillary line just behind the hairline. The incision should not extend inferiorly below the zygomatic arch or more than 2 cm anterior to the tragus to avoid injuring temporal branches of the facial nerve (TBFN) (Figure 2B-2C). In case a bypass is necessary during the procedure, careful dissection of the STA should be undertaken to preserve the trunk and its major branches.

Dissection of Scalp Flap

**Figure 3:**
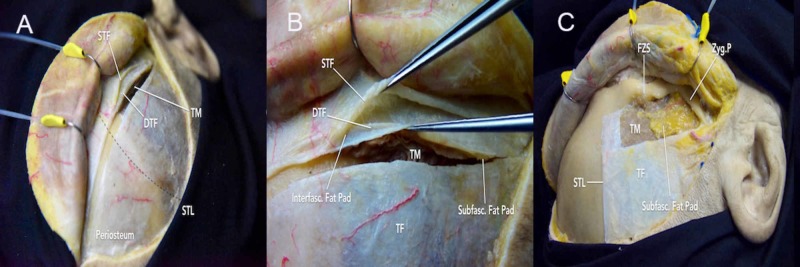
Fascial dissection (A) incision across superficial temporal fascia (inferior to STL) and frontal pericranium (superior to STL); (B) superficial layers, including the interfascial fat pad; (C) incision continued anteriorly to the lateral orbital rim. The dissection plane is maintained between the temporal fascia and temporalis muscle to elevate both fascial layers with the fat pad DTF = deep temporal fascia; FZS = frontozygomatic suture; Interfasc = interfascial; Subfasc = subfascial; STF=superior temporal fascia; STL = superior temporal line; TF = temporal fascia; TM = temporalis muscle

The skin, subcutaneous tissue, and galea are carefully dissected and elevated together over the orbit. Similar to the PA, transareolar (subgaleal) dissection is performed from the midline to the inferior portions until the suprafascial fat pad is observed. To protect TBFN that runs through the suprafascial fat pad, the underlying superficial temporal fascia should be elevated with the scalp flap in the subfascial plane [2,10]. The intrafascial dissection technique is used to incise the superficial temporal fascia from the most anterior part of STL to the inferior margin of the skin incision (the posterior root of the zygomatic arch) (Figure 3A-3C) [11,12,13]. Dissection should continue on the deep temporal fascia, below the fat pad, to elevate the superficial temporal fascia and fat pad over the zygomatic arch.

**Figure 4:**
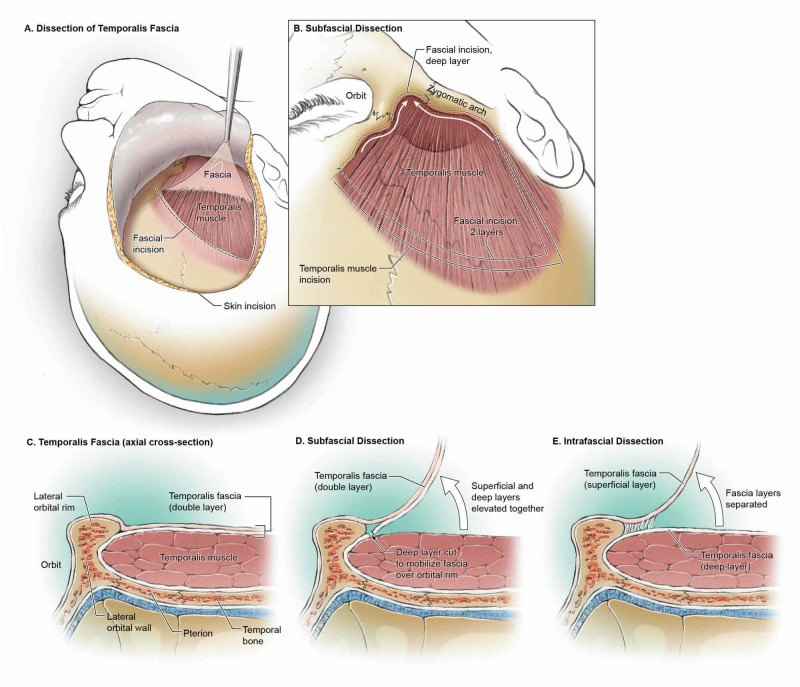
Overview of the intrafascial and subfascial dissections (A-B) after performing an intrafascial or subfascial dissection, one of the critical steps is the detachment of the layers of fascia from the zygoma and orbital rim. (C-E) an axial view of the topography can provide a better understanding of the region. While the superficial layer typically continues with the periosteum, the deep layer tends to attach to the inferior border of the bone; therefore, during a subfascial dissection, an incision of the fascia just above the muscle is necessary to release the flap and to expose the orbitozygomatic unit

Alternatively, the subfascial dissection technique can be used to elevate the superficial temporal fascia, fat pad, and deep temporal fascia together in a single step [12,13]. The superficial temporal fascia is incised along the skin incision and STL and continued anteriorly to the lateral orbital rim. The dissection plane is maintained between the temporal fascia and temporalis muscle to elevate both fascial layers with the fat pad. Furthermore, an additional cut on the deep temporal fascia is necessary to expose the zygomatic arch and lateral orbital rim (Figure 3C). This technique is simple, efficient, and it significantly minimizes the risk of injury to TBFN (Figure 4A-4E).

Mobilization of the Temporalis Muscle

**Figure 5:**
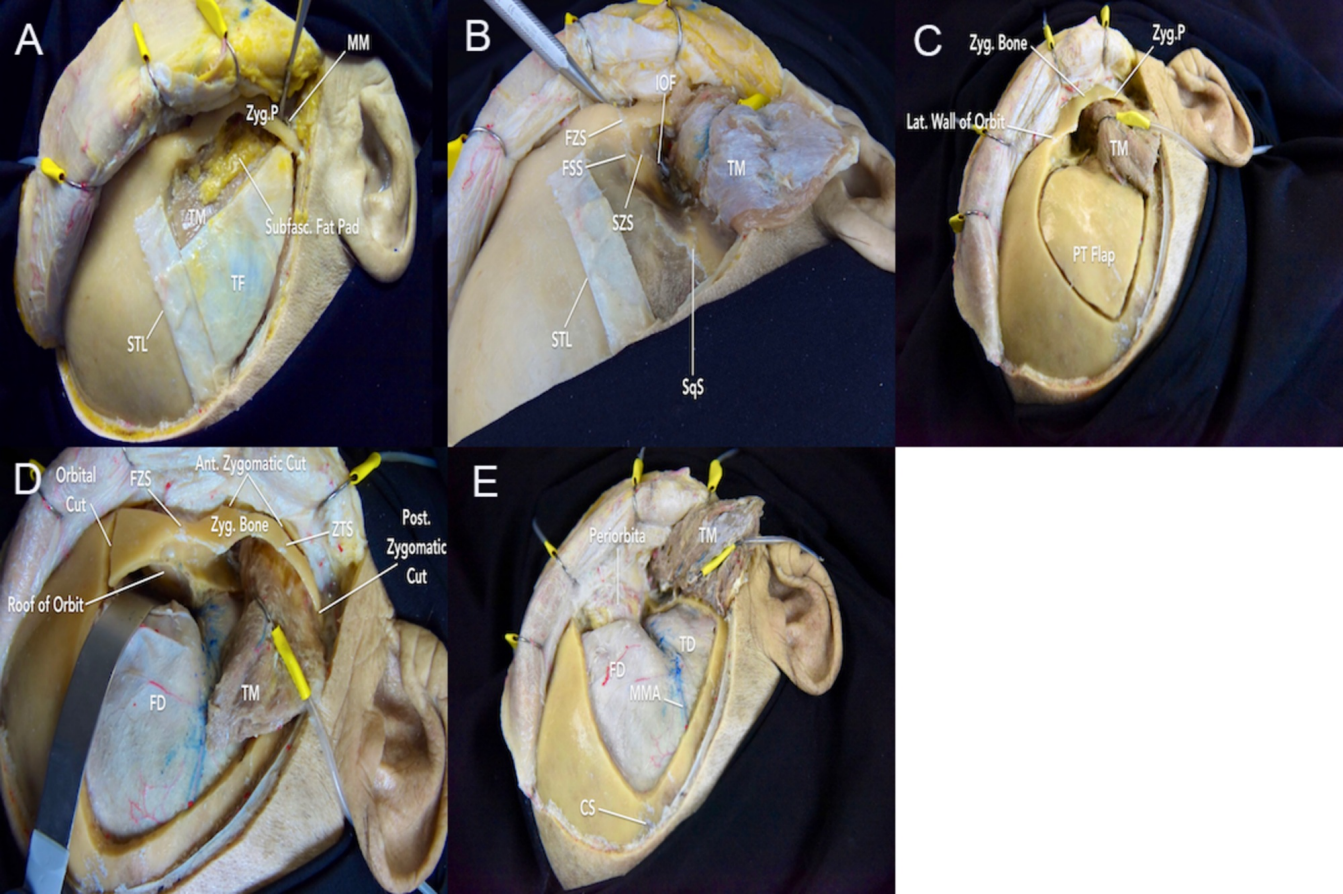
Two-piece frontotemporal-orbitozygomatic (FTOZ) approach A) further dissection of the deep temporal fascia facilitates exposure of the zygomatic arch and lateral orbital rim (steps that aid in the identification and isolation of delicate facial nerve branches); B) the temporalis muscle is dissected and elevated inferiorly, and the periorbita is gently dissected off orbital walls (the dissector is denoting the inferior orbital fissure); C) the first bone piece involves drilling the standard pterional bone flap; D) after removing the pterional bone flap, an orbital cut, anterior zygomatic cut, and posterior zygomatic cut are performed to remove the second bone piece; E) exposure of the frontal and temporal dura and periorbita following the two-piece FTOZ craniotomy CS = coronal suture; FD = frontal dura; FSS = frontosphenoid suture; FZS = frontozygomatic suture; IOF = inferior orbital fissure; MM = masseter muscle; MMA = middle meningeal artery; PT = pterional; SqS = squamosal suture; STL = superior temporal line; SZS = sphenozygomatic suture; TD = temporal dura; TF = temporal fascia; TM = temporalis muscle; ZTS = zygomaticotemporal suture; Zyg. = zygomatic; Zyg.P. = zygomatic process

The temporalis muscle is dissected from the inferior aspect of the skin incision and anteriorly to the inferior temporal line, leaving a musculofascial cuff along the STL for reattachment of the muscle during closure (Figure 5A). By dissecting in the opposite direction from the muscle fibers without using monopolar cautery, this retrograde technique reduces muscle atrophy and preserves the subperiosteal and deep temporal nerves and arteries. However, special attention should be given to avoid inadvertent damage to the middle temporal artery, the proximal medial branch of STA, which runs in close proximity to the zygomatic root. After dissection, the temporalis muscle is elevated inferiorly over the zygomatic arch in the subperiosteal plane to expose the middle cranial fossa floor.

Dissection of Periorbita

With the elevation of the temporal fascia, subperiosteal dissection can be performed along the lateral orbital rim and extended inferiorly to increase the exposure of the zygoma. It is important to maintain the dissection in the subperiosteal plane to avoid injuring distal branches of the frontalis nerve. The continuity of the periosteum and periorbita should be maintained across the superior orbital rim. From the lateral orbital rim, the periorbita is gently dissected from the superior and lateral walls of the orbit until the anterolateral portion of the IOF is reached (Figure 5B). Utmost care should be taken particularly near the superolateral aspect, where the lacrimal gland lies within the lacrimal fossa of the frontal bone and the periorbita is most adherent at the frontozygomatic junction. In addition, dissection can be continued medially toward the supraorbital notch/foramen to free the supraorbital nerve. At this stage, there should be complete exposure of the zygoma and orbital rim.

Craniotomy

The two-piece FTOZ involves the removal of the standard pterional bone flap, followed by the orbitozygomatic bone flap (Figure 5C, Interactive Model 4, Video 1). The first burr hole is drilled superior to the zygomatic arch in the temporal squamosal bone. The second burr hole should be placed at the MacCarty keyhole, which has been described to be found at mean distances of 6.8 mm superior and 4.5 mm posterior to the frontozygomatic suture. The MacCarty keyhole exposes the frontal lobe dura in the upper half and periorbita in the lower half of the keyhole with the orbital roof in between [14]. Accurate placement of the keyhole is critical for an efficient craniotomy that optimally preserves the lateral orbital wall and orbital roof. However, the number of burr holes can vary depending on the individual age and pathology of the patient. The bone cut can also be extended to the frontal region for anterior fossa lesions and inferiorly to the temporal region for middle and posterior fossae lesions. 

For the orbitozygomatic craniotomy, six bone cuts can be performed using the IOF and SOF as landmarks (Interactive Model 5). To protect the periorbital and intraorbital contents, thin-blade retractors or surgical patties can be used. The first slightly oblique cut is made across the posterior root of the zygomatic arch, just anterior to the articular tubercle of the glenoid fossa to avoid the risk of postoperative temporomandibular joint dysfunction. The second cut is made at the inferolateral margin of the zygomatic bone. The third cut is initiated in the anterolateral portion of the IOF and extends posterolaterally to join the second cut above the malar eminence. The IOF can be identified along the inferior portion of the lateral orbital wall. In preparation for the next cut, the dura is elevated from the superior and lateral walls of the orbit. The fourth cut is made perpendicular to the superior orbital rim and extends posteriorly across the orbital roof toward the SOF. While the orbital rim is a thick layer of bone, the orbital roof is relatively thin and must be carefully cut to prevent the release of periorbital fat and enophthalmos. The fifth and sixth cuts are made starting from the IOF and SOF, respectively, to join these structures and ultimately elevate the orbitozygomatic bone flap (Figure 5D-E). During the closure, the bone piece can be securely reattached with miniplates and screws. 

**Video 1:** Two-piece frontotemporal-orbitozygomatic (FTOZ) craniotomy The first bone piece involves a standard pterional bone flap. The second bone piece involves five bony cuts. The first cut is made at the posterior end of the zygomatic arch; the second cut is made across the inferolateral margin of zygoma; the third cut is made across the superior orbital rim and orbital roof to gently release the dura from periorbita; the fourth cut is made from the superior orbital fissure; the fifth cut is made from the inferior orbital fissure to connect to the fourth cut

**Video 4:** Volumetric model of the two-piece frontotemporal-orbitozygomatic (FTOZ) craniotomy Top left: a standard pterional bone flap is drilled using the MacCarty keyhole. Top right: the pterional bone flap is removed to expose the dura and inferior orbital fissure. Bottom left: five bony cuts are made to remove the second bone piece involving the lateral orbital rim and zygomatic root. Bottom right: exposure of the periorbita and inferior orbital fissure following removal of the second bone piece

**Video 5:** Volumetric model of the six bone cuts involved in the two-piece frontotemporal-orbitozygomatic (FTOZ) craniotomy The first cut is made across the posterior root of the zygomatic arch (blue line). The second cut is made at the inferolateral margin of the zygomatic bone (sky-blue line). The third cut is initiated in the anterolateral portion of the IOF and extends posterolaterally to join the second cut above the malar eminence (orange line). The fourth cut is made perpendicular to the superior orbital rim and extends posteriorly across the orbital roof toward the SOF (red line). The fifth and sixth cuts are made starting from the IOF (purple line) and the SOF (green line), respectively, to join these structures and ultimately elevate the orbitozygomatic bone flap

**Figure 6:**
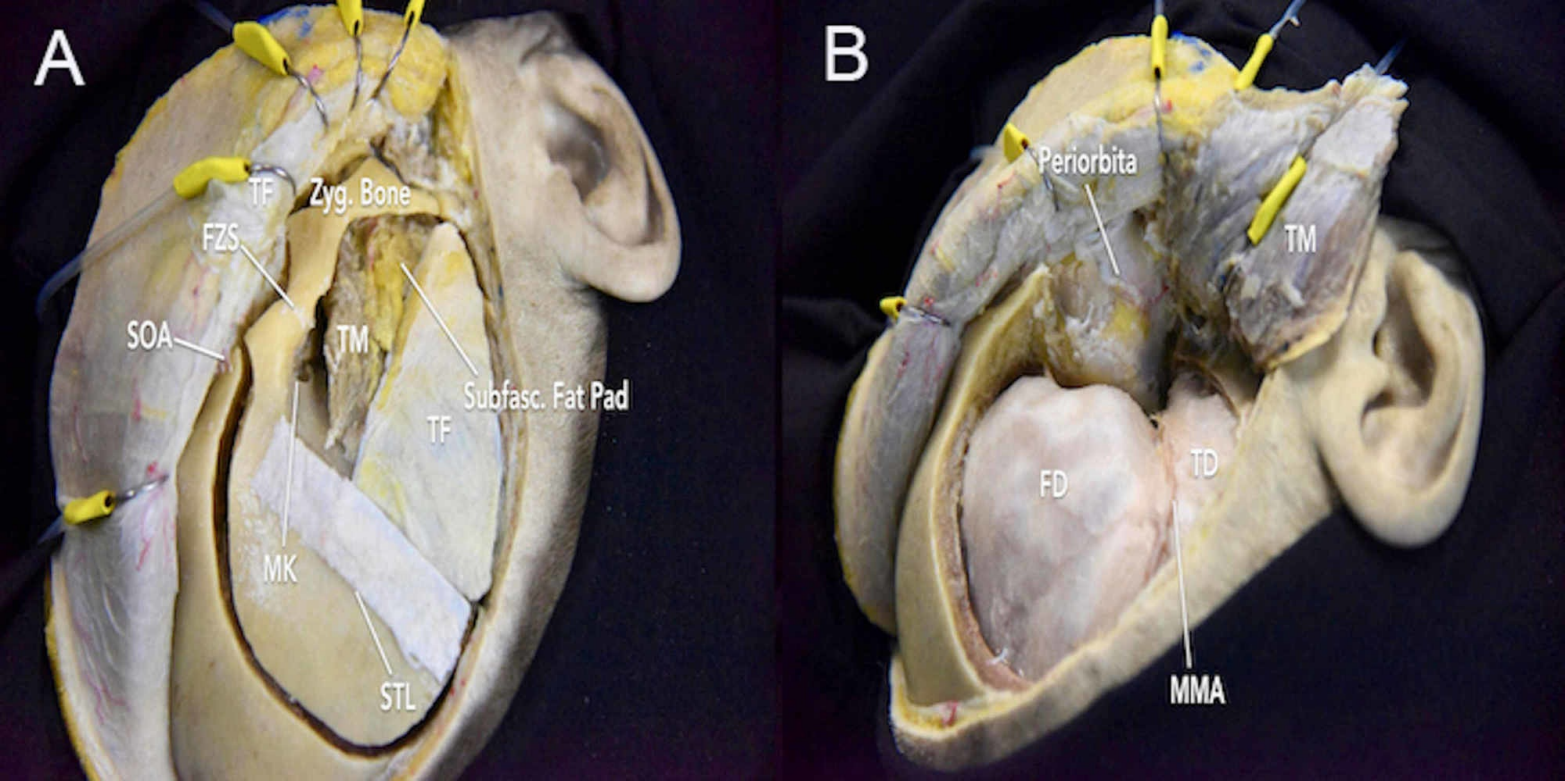
One piece frontotemporal-orbitozygomatic (FTOZ) approach (A) connecting cuts following the placement of the three required burr holes; (B) window of exposure following the one-piece FTOZ approach FD = frontal dura; FZS = frontozygomatic suture; MK = MacCarty keyhole; MMA = middle meningeal artery; SOA = superior orbital artery; STL = superior temporal line; Subfasc.Fat Pad = subfascial fat pad; TD = temporal dura; TF = temporal fascia; TM = temporalis muscle; Zyg.Bone = zygomatic bone

In the early 1980s, Jane et al. presented the one-piece FTOZ as an efficient method for reaching deep lesions in the skull base and orbit without extensive bony reconstruction [15]. In this method, three burr holes can be placed. The first burr hole is drilled in the frontal bone superior to the nasion. The second burr hole is placed at the MacCarty keyhole. The third burr hole is drilled posteriorly in the temporal region to expose the temporal lobe dura. Using the craniotome, the first and third burr holes are connected through an upward arc that reaches about 5 cm above the superior orbital rim (Video 2). Next, the second and third burr holes are connected with the craniotome passing above the temporal fossa. Finally, the first and second burr holes are connected by using a Gigli saw to incise the orbital roof and lateral orbital rim. Therefore, the one-piece FTOZ flap includes the superior and lateral orbital rim, parts of the orbital roof, and frontal and temporal bones (Figure 6A-B, Interactive Model 6). 

**Video 2:** One-piece frontotemporal-orbitozygomatic (FTOZ) craniotomy The one-piece FTOZ approach involves drilling the temporal burr hole and MacCarty keyhole to expose the periorbita and frontal dura. Four bony cuts are made. The first cut starts at the temporal burr hole and extends posteriorly above the superior temporal line to the superior orbital rim; the second cut connects the temporal burr hole to MacCarty keyhole; the third cut is made across the posterior roof the zygomatic arch; the fourth cut is made across the inferolateral margin of the zygoma and frontozygomatic process

**Video 6:** Volumetric model of bone flap removed in one-piece frontotemporal-orbitozygomatic (FTOZ) approach Four bony cuts are made to generate this bone flap. The first cut starts at the temporal burr hole and extends posteriorly above the superior temporal line to the superior orbital rim; the second cut connects the temporal burr hole to MacCarty keyhole; the third cut is made across the posterior roof the zygomatic arch; the fourth cut is made across the inferolateral margin of zygoma and frontozygomatic process

The one-piece FTOZ has been noted to reduce surgical time and achieve good cosmetic results. However, this technique provides limited exposure of the inferior orbital fissure due to the temporalis muscle mass. While Shigeno et al. refined the technique by transposing the temporalis muscle underneath the zygomatic arch, this requires a more inferior skin incision at the level of the zygomatic arch, which stands to increase the risk of injury to TBFN [16]. With a smaller removal of the orbital roof, the one-piece FTOZ craniotomy can also lead to reduced visualization of the basal frontal lobe.

**Figure 7:**
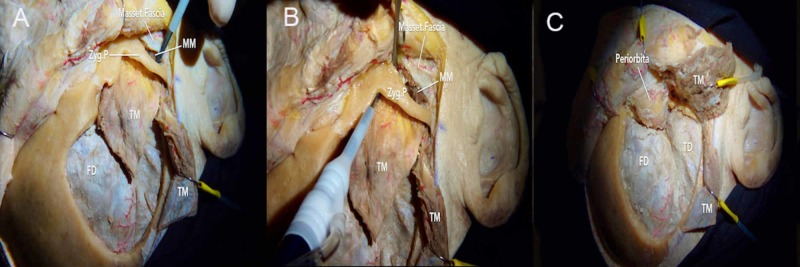
Three-piece frontotemporal-orbitozygomatic (FTOZ) approach (A) after removing the pterional bone flap, the masseter muscle is detached; (B) posterior and anterior cuts are made to remove the zygomatic arch; (C) exposure of the frontal and temporal dura following the three-piece FTOZ craniotomy FD = frontal dura; MM = masseter muscle; Masset.Fascia = masseter fascia; TM = temporalis muscle; Zyg.P = zygomatic process

Campero et al. popularized the three-piece FTOZ to obtain better exposure of the middle cranial fossa floor, lateral orbital rim, and the IOF [17]. The first bone piece is the standard pterional bone flap. The second bone piece involves the zygomatic arch, which is vertically cut at two ends: 1) anterior cut that is posterior to the zygomaticotemporal suture, and 2) posterior cut that is anterior to the temporomandibular joint (Video 3). This allows the zygomatic arch to be reclined inferiorly along with its attached masseter muscle. The temporalis muscle can then be reflected in this space to expose the middle cranial fossa floor. The orbital roof and lateral wall of the orbit are additionally drilled in preparation for removing the third bone piece, which involves the orbital rim, orbital roof, and lateral wall of the orbit (Figure 7A-C). Three cuts are performed in this region to release the orbitozygomatic bone flap, as described above in the standard two-piece FTOZ. However, the orbital roof cut should be extended around the SOF and directed toward the IOF to avoid neurovascular damage in the lateral part of the SOF. Compared to the one-piece and two-piece FTOZ, the three-piece FTOZ approach involves a similar amount of surgical time and postoperative complications (e.g., lesions of TBFN, supraorbital nerve, tissue edema).

**Video 3:** Three-piece frontotemporal-orbitozygomatic (FTOZ) craniotomy The first bone piece involves the zygomatic arch, which is vertically cut at two ends: 1) anterior cut that is posterior to the zygomaticotemporal suture, and 2) posterior cut that is anterior to the temporomandibular joint. The second bone piece is the standard pterional bone flap. The orbital roof and lateral wall of the orbit are additionally drilled in preparation for removing the third bone piece, which involves the orbital rim, orbital roof, and lateral wall. Three cuts are performed in this region to release the orbitozygomatic bone flap, as described in the standard two-piece FTOZ

Drilling of Basal Structures

The lesser wing of sphenoid, orbital roof and squamous temporal bone should be drilled to flatten the bone and increase access to basal areas. First, bony prominences on the orbital roof should be carefully flattened without inadvertently exposing the periorbita. Then, the lesser wing of the sphenoid can be drilled until the MOB is observed. Drilling beyond this medial limit will cause damage to the anterior clinoid process. To obtain the temporopolar view in the surgical field, the lateral portion of the greater wing of the sphenoid should be drilled until the temporal pole is observed. To obtain the subtemporal view, the floor of the middle cranial fossa should be drilled until the foramen spinosum is reached. This will facilitate anterior and basal detachment of the temporal lobe.

Dural Opening

The dura mater is cut through a C-shaped incision along the medial superior orbital margin and midportion of the inferior temporal region. This exposes the inferior frontal gyrus, part of the middle frontal gyrus, and the superior, middle, and inferior temporal gyri. The transsylvian approach is then followed to open the sylvian fissure and expose the basal cisterns.

## Discussion

The Mini-Orbitozygomatic Approach

**Figure 8:**
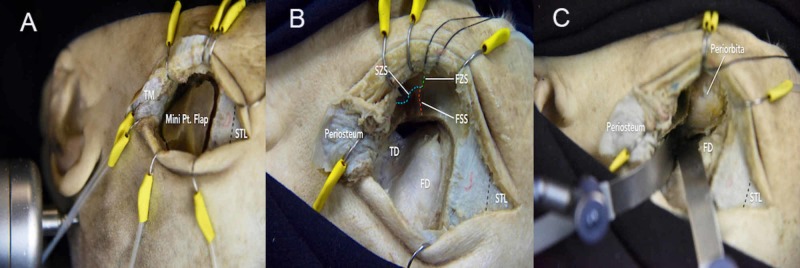
Mini-orbitozygomatic (MOz) approach (A) a curvilinear skin incision starts from 1 cm anterosuperior to the tragus and extends superiorly to the midpupillary line. A single burr hole is placed at the MacCarty keyhole on the surface of the temporal squamous bone, and a 3x3-cm craniotomy is performed; (B) the bone flap is removed to expose the temporal lobe dura; (C) the lesser wing of the sphenoid, orbital roof, and squamous temporal bone is drilled to flatten the bone and increase access to basal areas FD = frontal dura; FSS = frontosphenoidal suture; FZS = frontozygomatic suture; Pt = pterional; STL = superior temporal line; SZS = sphenozygomatic suture; TD = temporal dura; TM = temporal muscle

The mini-orbitozygomatic (MOz) approach has been commonly used to treat anterior communicating artery, internal carotid artery bifurcation, and superior cerebellar artery aneurysms [18]. The MOz approach starts with a curvilinear skin incision from 1 cm anterosuperior to the tragus and extends superiorly to the midpupillary line. After carrying out an intrafascial or subfascial dissection, a single burr hole is placed at the MacCarty keyhole on the surface of the temporal squamous bone. After releasing the periorbita from the superior and lateral orbital wall, a 3x3-cm craniotomy is performed (Video 4, Figure 8A-C). While the MOz approach provides a smaller craniotomy than the standard FTOZ approach, there can be comparable surgical exposure depending on the accurate placement of the keyhole craniotomy (Interactive Model 7). In addition, the MOz approach reduces tissue damage and atrophy of the temporalis muscle, which may improve cosmetic outcomes. 

**Video 4:** Mini-orbitozygomatic (MOz) craniotomy After removing the pterional bone flap, the orbital bone piece is drilled on the orbital roof. The orbital bone piece is removed to expose the periorbita

**Video 7:** Volumetric model of the two-piece frontotemporal-orbitozygomatic (FTOZ) (right side) and mini-orbitozygomatic (MOz) (left side) craniotomies The mini-orbitozygomatic (MOz) approach provides a smaller craniotomy that could be used to treat anterior communicating artery, internal carotid artery bifurcation, and superior cerebellar artery aneurysms

The Orbito-Pterional Approach

The orbito-pterional (OPt) approach is extensively reported in the literature for enabling the approach to the anterior communicating artery complex with wide working angles [19]. The surgical technique proceeds in a similar way, with one burr hole placed at the MacCarty keyhole and the other placed superior to the zygomatic arch in the temporal squamosal bone (temporal keyhole). We describe the one-piece version of the OPt approach as a simple technique that minimizes bony reconstruction at closure and reduces the risk of enophthalmos and cosmetic defects. The first bony cut begins at the temporal keyhole and arcs superiorly toward the supraorbital foramen. The second bony cut connects the temporal keyhole with the MacCarty keyhole. The third bony cut is made from the MacCarty keyhole to the anterolateral portion of the IOF. The fourth bony cut extends from the anterolateral portion of the IOF across the lateral orbital wall. The fifth bony cut is an anterior extension of the first cut into the orbital cavity. Finally, the sixth bony cut connects the fifth cut to the MacCarty keyhole along the orbital roof (Interactive Model 8).

**Video 8:** Volumetric model of orbito-pterional (OPt) window This approach provides wide access to the anterior communicating artery complex while preserving the zygomatic process

Compared to the standard FTOZ approach, the OPt approach is less invasive as the zygomatic arch is not removed. Moreover, Schwartz et al. have suggested that the additional extent of exposure provided by removing the zygomatic arch is considerably less compared to removing the orbital rim [20]. Therefore, the OPt approach may often be sufficient for deeper and higher lesions. In any case, the selection of surgical technique should be based on the unique pathology and needs of the patient. 

## Conclusions

The FTOZ approach is one of the workhorse techniques in neurosurgery that provides wide exposure and improved access to the cavernous sinus and anterior, middle, and posterior cranial fossae. Similar to the PA, the FTOZ approach involves carefully preserving TBFN during fascial dissection. Additionally, knowledge of the structures surrounding the orbit and zygoma is critical for adopting the FTOZ approach. In particular, the SOF and IOF are important landmarks that must be referenced during the FTOZ craniotomy.
